# Effect of Ultra-Fast Light Curing on the Irradiated-Surface Degree of Conversion and Polymer-Network Quality of Bioactive and Conventional Bulk-Fill Resin Composites: An In Vitro Study

**DOI:** 10.3390/ma19153323

**Published:** 2026-08-05

**Authors:** Sarah Fahad Alsenani, Sultan Binalrimal

**Affiliations:** Department of Restorative Dentistry, College of Medicine and Dentistry, Riyadh Elm University, Riyadh 12734, Saudi Arabia; sara.f.alsenani@gmail.com

**Keywords:** bulk-fill composite, degree of conversion, polymer network quality, ethanol softening, ultra-fast curing, bioactive composite, giomer

## Abstract

**Highlights:**

- Ultra-fast curing affected irradiated-surface polymerization in a material-dependent manner.
- Bioactive and conventional bulk-fill composites exhibited distinct polymer-network responses.
- Degree of conversion and polymer-network quality provided complementary information.
- Composite formulation influenced network quality more than curing protocol.
- Ultra-fast curing should be validated individually for each restorative material.

**Abstract:**

**Background:** Ultra-fast high-irradiance curing shortens exposure time, but its effects on conversion and polymer-network integrity may vary among bulk-fill resin composites. This in vitro study was designed as a materials-level investigation of irradiated-surface polymerization behavior. It evaluated irradiated-surface degree of conversion and polymer-network quality while deliberately excluding assessment of depth of cure or polymerization throughout the 4 mm bulk increment. **Methods:** Eighty disk specimens (6 mm × 4 mm) of Filtek One Bulk Fill, SDR Plus, Beautifil Bulk Flowable, and Beautifil Bulk Restorative (n = 10/material/protocol) were cured with a polywave LED at 1200 mW/cm^2^ for 10 s or 3000 mW/cm^2^ for 3 s. Degree of conversion (DC) was measured by ATR-FTIR on the irradiated (top) surface only. Ethanol-induced softening (ES%, Knoop-hardness reduction at the irradiated surface after 24 h ethanol immersion) served as an inverse, indirect indicator of polymer-network quality (cross-link density). Data were analyzed by two-way ANOVA and Tukey tests (α = 0.05); the DC–ES% relationship was examined by Pearson correlation at the individual-specimen level (n = 80). **Results:** Ultra-fast curing yielded higher pooled DC (66.30 ± 10.06%) than conventional curing (59.37 ± 7.96%; *p* < 0.001), but the effect was material-dependent: DC increased significantly for Filtek One Bulk Fill (*p* < 0.001) and Beautifil Bulk Flowable (*p* = 0.035), but not for SDR Plus or Beautifil Bulk Restorative. ES% was influenced mainly by material (*p* < 0.001) and less by curing protocol (*p* = 0.009). At the specimen level (n = 80), DC and ES% showed a weak but statistically significant negative correlation (r = −0.31, *p* = 0.006). **Conclusions:** Within the conditions of this study, irradiated-surface polymerization behavior under ultra-fast curing depended primarily on composite formulation, underscoring the need to evaluate both conversion efficiency and polymer-network quality when optimizing dental restorative materials. Because conversion was assessed only at the irradiated surface, these findings describe irradiated-surface behavior and do not provide evidence of adequate cure at the bottom of the 4 mm increment.

## 1. Introduction

Resin-based composites are the predominant direct restorative materials because they combine esthetics with adhesive, tooth-preserving treatment. Their clinical performance depends partly on the quality of polymerization, which is governed by resin chemistry, filler architecture, photoinitiator systems, and the delivered light exposure [1,2,3].

Bulk-fill composites were developed to simplify posterior restoration by permitting increments of approximately 4–5 mm. Increased translucency, modified monomer systems, and optimized photoinitiators facilitate light transmission and polymerization at greater depths; however, substantial product-to-product variation remains [2,4,5]. Contemporary bulk-fill materials include sculptable and flowable formulations, as well as giomer-based materials containing surface pre-reacted glass-ionomer (S-PRG) fillers that can release multiple ions and are marketed as bioactive [6,7].

The degree of conversion (DC) describes the proportion of methacrylate carbon-carbon double bonds converted during polymerization. Higher conversion is generally associated with improved mechanical behavior and reduced residual-monomer elution, although DC alone does not fully describe the architecture of the polymer network [8,9]. Cross-linking and network heterogeneity influence solvent resistance, hardness, and long-term stability. Solvent-softening tests therefore provide complementary information by evaluating the resistance of the cured resin matrix to plasticization [10,11,12].

High-power polywave light-curing units have enabled exposure protocols as short as 3 s. These protocols may improve clinical efficiency, but the exposure reciprocity relationship may not hold at very high irradiance. Rapid radical generation can restrict monomer mobility and promote early vitrification or heterogeneous network formation, making the response dependent on the material and its photoinitiator chemistry [13,14,15,16,17]. Previous studies have reported both acceptable and compromised polymerization after ultra-fast curing, emphasizing that such protocols should not be generalized across products [14,15,16,17,18,19,20].

Limited evidence directly compares ultra-fast and conventional curing across conventional and bioactive (giomer, S-PRG-containing) bulk-fill formulations while evaluating both DC and polymer-network quality. Clarifying these irradiated-surface polymerization responses improves understanding of material-dependent behavior under accelerated curing protocols. Therefore, this study characterized the irradiated-surface degree of conversion and polymer-network quality of four bulk-fill resin composites, including two giomer-based bioactive materials, under conventional (1200 mW/cm^2^ for 10 s) and ultra-fast (3000 mW/cm^2^ for 3 s) curing. The investigation was deliberately confined to the irradiated surface as a materials-level comparison and does not evaluate the depth of cure or the polymerization of the 4 mm increment as a whole; the 4 mm specimen thickness was intentionally selected to reproduce the clinically recommended bulk-fill increment geometry and the optical pathway encountered during clinical light curing, while measurements were deliberately confined to the irradiated surface. The null hypotheses were that the curing protocol would not affect (1) DC or (2) polymer-network quality, and that (3) DC would not be associated with polymer-network quality.

## 2. Materials and Methods

### 2.1. Study Design and Ethical Approval

An in vitro factorial experiment evaluated four bulk-fill resin composites under two light-curing protocols. The primary outcomes were irradiated surface degree of conversion and polymer-network quality assessed indirectly using ethanol-induced softening. The study was designed as a materials-level investigation of irradiated-surface polymerization behavior; depth of cure was not assessed. The study protocol was reviewed and approved by the Institutional Review Board of Riyadh Elm University (approval no. FPGRP/2025/935/1332/1204). Because the study did not involve humans or animals, informed consent was not applicable.

### 2.2. Materials

Four commercially available materials representing conventional and giomer-based bioactive bulk-fill technologies were investigated (Table 1). Compositional information was obtained from manufacturer documentation and the literature.

### 2.3. Specimen Preparation and Allocation

Disk-shaped specimens were prepared in custom cylindrical molds (6 mm diameter × 4 mm thickness). Each material was inserted as a single bulk increment. The 4 mm thickness was selected because it corresponds to the maximum single-increment depth claimed by the manufacturers of all four bulk-fill materials; specimens therefore reproduced the clinically intended material geometry and the incident light-delivery conditions at the irradiated surface of a bulk increment. Measurement was deliberately confined to this irradiated surface to characterize the material- and protocol-dependent polymerization response at the irradiated surface, where polymerization is generally expected to be greatest, whereas depth-dependent light attenuation, a distinct phenomenon governed largely by translucency and filler scattering, was outside the scope of this study. This design should not be interpreted as implying that irradiated-surface polymerization alone is sufficient to characterize the clinical curing performance of bulk-fill resin composites. A Mylar strip and glass slide were placed over the material to minimize the oxygen-inhibited layer, flatten the surface, and standardize thickness. Specimens were allocated to eight groups according to material and curing protocol (10 specimens per group; total n = 80; Table 2), and the sequence of specimen preparation and measurement was randomized using a computer-generated random order. Conventional curing groups served as the reference; no separate external control material was included.

### 2.4. Light-Curing Procedures

A Bluephase PowerCure polywave LED light-curing unit (Ivoclar Vivadent, Schaan, Liechtenstein; wavelength range 385–515 nm) was used for both protocols. Conventional curing was performed at 1200 mW/cm^2^ for 10 s, whereas ultra-fast curing was performed at 3000 mW/cm^2^ for 3 s. The light guide was positioned perpendicular to and in contact with the Mylar strip. Irradiance was verified before the experiment and monitored periodically using a Bluephase Meter II radiometer (Ivoclar Vivadent). The radiometer-measured outputs were (1190 ± 13 mW/cm^2^) for the conventional setting and (2995 ± 6 mW/cm^2^) for the ultra-fast setting, both within the manufacturer’s stated tolerance of the nominal values. The corresponding nominal radiant exposures were approximately 12 J/cm^2^ (conventional) and 9 J/cm^2^ (ultra-fast). The conventional protocol is consistent with the manufacturers’ instructions for use for all four materials, whereas the 3 s ultra-fast protocol corresponds to the dedicated high-output mode of the curing unit and is not part of the validated instructions of every material tested; it was included because high-output units make a 3 s cure clinically available for materials not individually validated for it.

### 2.5. Degree of Conversion

Degree of conversion was measured using attenuated total reflectance Fourier-transform infrared spectroscopy (ATR-FTIR). For each specimen, the spectrum of the uncured composite was recorded on the top (irradiated) surface in the mold, followed by acquisition of the post-curing spectrum of the same surface immediately after the end of light exposure (within 30 s). Identical timing was applied to all specimens, so the reported values represent the immediate post-cure degree of conversion; any subsequent post-cure (dark) polymerization affected all groups equally. The aliphatic C=C absorption band at 1640 cm^−1^ was normalized to the aromatic C=C reference band at 1608 cm^−1^. DC was calculated as DC (%) = [1 − (Rpolymerized/Runpolymerized)] × 100, where R is the ratio of the aliphatic to aromatic peak intensity [8]. Conversion was assessed on the irradiated surface only; bottom-surface conversion at the 4 mm depth was not measured.

### 2.6. Ethanol-Induced Softening (Indirect Assessment of Polymer-Network Quality)

After FTIR assessment, specimens were removed from the molds and stored dry, in darkness, at room temperature (23 ± 1 °C) for 24 h until hardness testing. For each specimen, three Knoop indentations were made on the irradiated surface at standardized locations (load 50 g, dwell 15 s) and averaged to yield a single specimen-level Knoop hardness value. Baseline Knoop microhardness was measured for each specimen. Specimens were then immersed in absolute ethanol at room temperature for 24 h, after which Knoop microhardness was measured again using the identical indentation protocol. All hardness measurements were performed by an operator blinded to material and curing-protocol allocation using coded specimens. Ethanol-induced softening, expressed as the percentage reduction in Knoop hardness (ES%), was used as an inverse, indirect indicator of polymer-network quality (cross-link density); it is not a direct measurement of cross-link density and is additionally influenced by resin composition, filler content, filler–matrix interfaces, and solvent interaction. ES% was calculated as [(KHNinitial − KHNfinal)/KHNinitial] × 100, where KHNinitial and KHNfinal represent Knoop hardness before and after ethanol immersion, respectively. Lower ES% values indicate less ethanol-induced softening, greater solvent resistance, and therefore higher cross-link density, whereas higher ES% values indicate greater hardness reduction and lower cross-link density [10,11,12].

### 2.7. Statistical Analysis

Statistical analyses were conducted using SPSS Statistics version 29.0 (IBM Corp., Armonk, NY, USA) and GraphPad Prism version 10.0 (GraphPad Software, Boston, MA, USA). Means, standard deviations, minima, and maxima were calculated. An a priori power analysis (G*Power 3.1.9.7) indicated that 10 specimens per group provided at least 80% power to detect a large effect size (Cohen’s f = 0.40) for the main effects and the interaction of the two-way ANOVA at α = 0.05; this group size is also consistent with comparable in vitro studies of resin-composite polymerization. Two-way analysis of variance (ANOVA) tested the main effects of material and curing protocol and their interaction for DC and ES%. Tukey HSD tests were used for post hoc comparisons. The relationship between DC and ES% was examined by Pearson correlation of the individual specimen values (n = 80) rather than group means, in order to provide adequate statistical power; this analysis is presented as a supporting analysis and interpreted cautiously. Statistical significance was set at *p* < 0.05.

## 3. Results

### 3.1. Degree of Conversion

Across all materials, ultra-fast curing produced a higher mean DC (66.30 ± 10.06%) than conventional curing (59.37 ± 7.96%). The ultra-fast protocol also showed a wider range and greater variability (Table 3).

**Figure 1:**
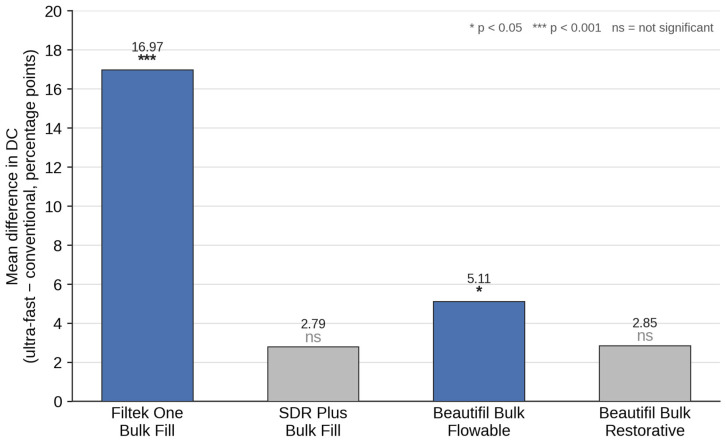
Mean within-material difference in degree of conversion (ultra-fast − conventional) for the four bulk-fill composites. * *p* < 0.05; *** *p* < 0.001; ns, not significant (Tukey HSD).

Two-way ANOVA identified significant effects of curing mode, material, and their interaction on DC (Table 4). The significant interaction indicated that the effect of ultra-fast curing differed among materials. Within-material Tukey comparisons showed significant increases for Filtek One (mean difference, 16.97 percentage points; *p* < 0.001) and Beautifil Bulk Flowable (5.11 percentage points; *p* = 0.035), but not for SDR Plus (2.79 percentage points; *p* = 0.638) or Beautifil Bulk Restorative (2.85 percentage points; *p* = 0.614). This material-dependent pattern is shown in Figure 1.

### 3.2. Polymer-Network Quality (Indirect Assessment)

**Figure 2:**
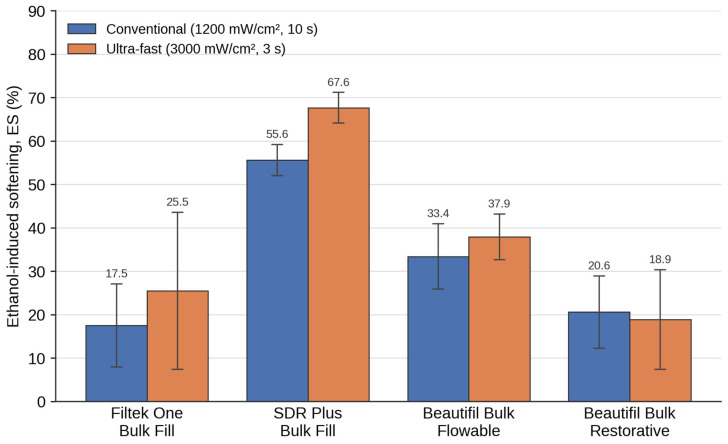
Ethanol-induced softening (ES%, mean ± standard deviation) of the four bulk-fill resin composites following conventional and ultra-fast curing. ES% is an inverse, indirect indicator of cross-link density: lower values indicate greater resistance to ethanol-induced softening and therefore higher cross-link density.

Figure 2 shows substantial differences in ethanol-induced softening among the four bulk-fill composites under both curing protocols. Because ES% represents the percentage reduction in hardness after ethanol immersion, lower values indicate greater cross-link density. Filtek One and Beautifil Bulk Restorative generally showed the lowest ES% values and therefore the greatest resistance to ethanol-induced softening, whereas SDR Plus showed the highest ES% values and therefore the lowest indirect cross-link density. Ultra-fast curing increased variability most prominently for Filtek One.

Two-way ANOVA showed a significant effect of material (*p* < 0.001) and a smaller significant effect of curing mode (*p* = 0.009), with no significant material × curing interaction (*p* = 0.144) (Table 5). At the specimen level (n = 80), DC and ES% showed a weak but statistically significant negative correlation (r = −0.31, *p* = 0.006). Higher irradiated-surface degree of conversion was therefore weakly associated with greater resistance to ethanol-induced softening; however, conversion explained less than 10% of the variance in ES% (r^2^ = 0.09), indicating that degree of conversion alone is a poor predictor of polymer-network quality.

## 4. Discussion

This investigation was designed as a materials-level comparison confined to the irradiated surface; the 4 mm specimen thickness reproduced the clinically intended increment geometry of these bulk-fill materials but was not used to evaluate depth of cure. The principal irradiated-surface finding was that the influence of ultra-fast curing on DC was strongly material-dependent. Although pooled DC was higher after 3 s curing at 3000 mW/cm^2^, the significant material × curing interaction shows that this overall mean should not be interpreted as universal superiority of ultra-fast curing. Filtek One showed the largest increase, Beautifil Bulk Flowable showed a smaller increase, and SDR Plus and Beautifil Bulk Restorative showed no significant protocol-related difference. Thus, the first null hypothesis was rejected.

This material dependence agrees with evidence that rapid curing outcomes are controlled not only by irradiance but also by radiant exposure, spectral compatibility, resin viscosity, filler loading, translucency, and polymerization-modulating monomers [13,14,15,16,17,18]. The conventional and ultra-fast protocols delivered nominal radiant exposures of approximately 12 and 9 J/cm^2^, respectively. Despite the lower nominal exposure, the ultra-fast protocol increased DC in selected materials, suggesting that formulation-specific kinetics and light transmission at the irradiated surface likely contributed to the observed differences. It is important to note that the two protocols differed not only in irradiance (1200 vs. 3000 mW/cm^2^) and exposure time (10 vs. 3 s) but also in total radiant exposure (≈12 vs. ≈9 J/cm^2^). Consequently, this study compares two specific, clinically used curing protocols and does not isolate the effect of increased irradiance alone; the observed differences cannot be attributed to high irradiance per se. This interpretation is consistent with previous reports that the duration and mode of photopolymerization jointly determine conversion and mechanical outcomes [21]. Because neither temperature nor polymerization kinetics was measured in this study, the following interpretation is offered as a hypothesis supported by prior work rather than as a confirmed mechanism. One possible explanation is the transient temperature rise associated with very high irradiance [22], which could increase radical and monomer mobility and thereby accelerate conversion. However, high irradiance may also create conversion gradients or network heterogeneity, which may explain the greater pooled variability observed with ultra-fast curing [13,15,16]. These mechanisms were not directly tested here and require dedicated real-time investigation.

Filtek One was the most responsive material in terms of DC. Its resin system contains AUDMA, UDMA, DDDMA, and an addition-fragmentation monomer. Addition-fragmentation chemistry can permit network rearrangement during curing and may support conversion under rapid high-intensity exposure [17,23]. Filtek One also showed relatively low mean ES% values, indicating comparatively high resistance to ethanol-induced softening, although variability was substantial, particularly after ultra-fast curing. This distinction is consistent with studies showing that conversion and cross-linking are related but non-equivalent descriptors of polymerization quality [9,10,11,12].

SDR Plus exhibited comparable DC under both protocols but showed the highest and most consistent ES% values. Because ES% represents percentage hardness reduction after ethanol immersion, these higher values indicate greater solvent-induced softening and therefore lower indirect cross-link density. Its modified UDMA-based resin and flowable consistency may facilitate radical mobility and conversion at the irradiated surface, but the resulting polymer network may remain more susceptible to ethanol plasticization. Previous reviews have found that flowable bulk-fill composites frequently achieve favorable conversion because of lower filler volume and enhanced light transmission [4,5].

The two giomer-based materials behaved differently despite sharing S-PRG technology. Beautifil Bulk Flowable showed a modest DC increase after ultra-fast curing and moderate variability in the solvent-softening outcome, whereas Beautifil Bulk Restorative showed no significant DC change and greater variability. The much higher filler loading of the restorative material may restrict light transmission and monomer mobility. Earlier work has also demonstrated product-specific conversion behavior for giomer bulk-fill composites [24]. These findings caution against treating all “bioactive” or S-PRG-containing materials as a homogeneous class.

Material type explained 77.4% of the variance in ES%, while curing mode explained 9.0%, and the interaction was not significant. This supports rejection of the second null hypothesis for the main effects but suggests a more consistent curing-mode influence across materials than was observed for DC. At the specimen level (n = 80), DC and ES% were weakly but significantly negatively correlated (r = −0.31, *p* = 0.006); the third null hypothesis was therefore rejected, although conversion explained less than 10% of the variance in ES%. This weak association indicates that higher surface conversion is only marginally related to greater solvent resistance and that degree of conversion alone remains a poor predictor of polymer-network quality. Because this correlation was computed at the individual-specimen level rather than from eight group means, it carries substantially greater statistical power than the original analysis while remaining a supporting analysis. Nevertheless, the present findings reinforce that high conversion does not necessarily guarantee a highly cross-linked or solvent-resistant polymer network [9,10,11,12].

Within the limits of irradiated-surface evaluation, ultra-fast curing demonstrated material-dependent responses. These findings alone should not be interpreted as supporting the adequacy of curing throughout a 4 mm bulk increment. The light-curing unit, tip positioning, exposure conditions, and material-specific manufacturer instructions remain essential. Ultra-fast modes should be used only when the restorative material has been validated for the relevant irradiance and exposure time [14,16,17,18,19,20,25,26].

Recent studies published in 2024 and 2025 further support a material-dependent response to accelerated curing. Contemporary investigations have shown that curing mode, filler content, depth, polymerization kinetics, and aging can alter microhardness and conversion outcomes, and that 3 s curing is most predictable in materials specifically engineered for rapid polymerization [19,20,26].

This study has limitations. It was conducted under controlled in vitro conditions and did not reproduce intraoral temperature, moisture, cavity geometry, or clinically relevant light-tip distances. The principal limitation is that both degree of conversion and hardness were evaluated only at the irradiated surface. Consequently, the present findings provide no evidence regarding polymerization at the bottom of the 4 mm increment. Future studies should therefore incorporate bottom-surface degree of conversion and the bottom-to-top microhardness ratio (ISO 4049 [27]) to evaluate depth-dependent polymerization. Only one light-curing unit and one specimen thickness were evaluated. This standardized top-surface approach was selected so that differences could be attributed to material and protocol rather than to depth-dependent light attenuation. The ethanol-softening method provides an indirect estimate of cross-link density and can be influenced by resin composition, filler-matrix interfaces, solvent concentration, and hardness-testing conditions. Accordingly, ES% should be interpreted as an inverse indirect indicator rather than a direct measurement of the number of cross-links. Future work should include real-time polymerization kinetics, direct or complementary network-characterization methods, depth-dependent measurements, aging, and mechanical testing. Direct characterization techniques such as dynamic mechanical analysis (DMA), Raman spectroscopy, or solvent swelling analysis could further validate the present indirect assessment of polymer-network quality.

## 5. Conclusions

This study evaluated only the irradiated surface polymerization behavior of the tested bulk-fill composites and did not assess the polymerization of the 4 mm increment as a whole. Within the limitations of this in vitro study, ultra-fast curing increased irradiated-surface degree of conversion in a material-dependent manner: significant increases were observed for Filtek One Bulk Fill and Beautifil Bulk Flowable, whereas SDR Plus and Beautifil Bulk Restorative showed no significant change. Material formulation was the dominant factor influencing resistance to ethanol-induced softening, and surface conversion was only weakly associated with ethanol-softening resistance at the specimen level (r = −0.31). Because ES% was calculated as percentage hardness reduction after ethanol immersion, lower values indicated greater indirect cross-link density. These findings are limited to irradiated-surface degree of conversion and ethanol-softening resistance; polymerization depth, mechanical properties, polymerization shrinkage stress, marginal integrity, aging, and clinical performance were not evaluated. Collectively, these findings support the need for material-specific validation of ultra-fast curing protocols, including assessment of depth of cure before broad clinical recommendations are made.

Clinically, the finding that conversion reached only about 59% to 66% even at the irradiated surface indicates that a substantial proportion of methacrylate double bonds remained unreacted. Residual monomers may elute into the oral environment and may plasticize the resin matrix, while unreacted pendant methacrylate groups may reflect a less densely cross-linked polymer network. Together, these factors may compromise long-term resistance to degradation and wear, with possible consequences for restoration durability. Because the response to ultra-fast curing differed among the composites tested, this protocol should be applied only to restorative materials for which it has been specifically validated, and conventional exposure times should be maintained otherwise. Future generations of resin composites should be engineered to achieve a higher degree of conversion under clinically realistic curing conditions through more efficient photoinitiator systems, optimized monomer chemistry, and improved light transmission, thereby enhancing the long-term clinical quality and durability of restorations.

## Figures and Tables

**Table 1:** Materials investigated in the study.

Material	Manufacturer	Material Class	Reported Composition
Filtek One Bulk Fill	3M ESPE, St. Paul, MN, USA	Conventional sculptable bulk-fill	AUDMA, AFM, UDMA, DDDMA; silica and zirconia/silica clusters; ytterbium trifluoride; 76.5 wt% (58.4 vol%) filler; CQ
SDR Plus Bulk Fill	Dentsply Sirona, Konstanz, Germany	Flowable bulk-fill	Modified UDMA resin, TEGDMA; barium-alumino-fluoro-borosilicate glass and silica; 68 wt% (45 vol%) filler; CQ
Beautifil Bulk Flowable	Shofu Inc., Kyoto, Japan	Giomer bioactive flowable bulk-fill	Bis-GMA, UDMA, TEGDMA; S-PRG filler and silica; 69 wt% (50 vol%) filler; CQ
Beautifil Bulk Restorative	Shofu Inc., Kyoto, Japan	Giomer bioactive sculptable bulk-fill	Bis-GMA, UDMA, TEGDMA; S-PRG glass and silica; 87 wt% (74 vol%) filler; CQ

AUDMA, aromatic urethane dimethacrylate; AFM, addition-fragmentation monomer; UDMA, urethane dimethacrylate; DDDMA, 1,12-dodecane dimethacrylate; TEGDMA, triethylene glycol dimethacrylate; Bis-GMA, bisphenol A glycidyl methacrylate; S-PRG, surface pre-reacted glass ionomer; CQ, camphorquinone.

**Table 2:** Experimental groups.

Group	Material	Curing Protocol	n
FCL	Filtek One Bulk Fill	Conventional, 1200 mW/cm^2^, 10 s	10
FUL	Filtek One Bulk Fill	Ultra-fast, 3000 mW/cm^2^, 3 s	10
SDCL	SDR Plus Bulk Fill	Conventional, 1200 mW/cm^2^, 10 s	10
SDUL	SDR Plus Bulk Fill	Ultra-fast, 3000 mW/cm^2^, 3 s	10
BFCL	Beautifil Bulk Flowable	Conventional, 1200 mW/cm^2^, 10 s	10
BFUL	Beautifil Bulk Flowable	Ultra-fast, 3000 mW/cm^2^, 3 s	10
BRCL	Beautifil Bulk Restorative	Conventional, 1200 mW/cm^2^, 10 s	10
BRUL	Beautifil Bulk Restorative	Ultra-fast, 3000 mW/cm^2^, 3 s	10

**Table 3:** Degree of conversion pooled across materials by curing protocol.

Protocol	n	Mean DC (%)	SD	Minimum	Maximum
Conventional	40	59.37	7.96	43.71	72.91
Ultra-fast	40	66.30	10.06	49.68	86.41

**Table 4:** Two-way ANOVA for degree of conversion.

Source	df	F	*p*-Value	Partial η^2^
Curing mode	1	77.77	<0.001	0.512
Material	3	130.70	<0.001	0.841
Curing × material	3	18.63	<0.001	0.428

**Table 5:** Two-way ANOVA for ethanol-induced softening (ES%).

Source	df	F	*p*-Value	Partial η^2^
Curing mode	1	7.12	0.009	0.090
Material	3	82.08	<0.001	0.774
Curing × material	3	1.86	0.144	0.072

## Data Availability

The original contributions presented in this study are included in the article/Supplementary Material. Further inquiries can be directed to the corresponding author.

## Supplementary Materials

The following supporting information can be downloaded at: https://www.mdpi.com/article/10.3390/ma19153323/s1, Figure S1: Representative specimen preparation and group photographs; Figure S2: Representative ATR-FTIR spectra; Figure S3: (A) Knoop indentation images—F and SD groups (B) Knoop indentation images—BF and BR groups.

## Abbreviations

The following abbreviations are used in this manuscript:

ATR-FTIR	Attenuated total reflectance Fourier-transform infrared spectroscopy
ES%	Ethanol-induced softening (percentage Knoop-hardness reduction)
CQ	Camphorquinone
DC	Degree of conversion
KHN	Knoop hardness number
S-PRG	Surface pre-reacted glass ionomer
