# Supplementary material for: Effect of Ultra-Fast Light Curing on the Irradiated-Surface Degree of Conversion and Polymer-Network Quality of Bioactive and Conventional Bulk-Fill Resin Composites: An In Vitro Study
Source: Materials (Basel). 2026 Aug 5;19(15):3323. doi: 10.3390/ma19153323 (PMC13468653; doi:10.3390/ma19153323)
Supplement: Supplementary file 1 [file materials-19-03323-s001.zip › materials-4437839-supplementary.pdf]

Supplementary figures

Effect of Ultra-Fast Light Curing on the Degree of Conversion and Polymer-Network Quality of Bioactive and Conventional Bulk-Fill Resin Composites: An In Vitro Study

Contents

- Figure S1. Representative specimen preparation, group labeling, and curing verification photographs.
- Figure S2. Representative ATR-FTIR spectra before and after light curing.
- Figure S3. Representative optical images of Knoop indentations before and after 24 h ethanol immersion for the eight experimental groups.

Prepared for the revision response to Reviewer 3, documenting the specimens, FTIR spectra, and Knoop indentation images underlying the reported values.

Figure S1. Representative specimen preparation and group photographs

A. Specimen fabrication in custom mold	B. Specimen / ATR-FTIR positioning	C. Disk thickness verification (4.0 mm)	D. Radiometer output — ultra-fast setting
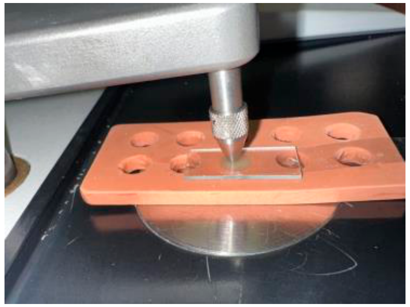	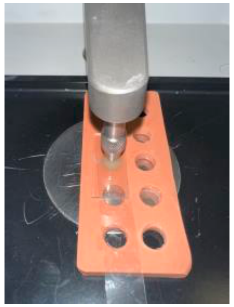	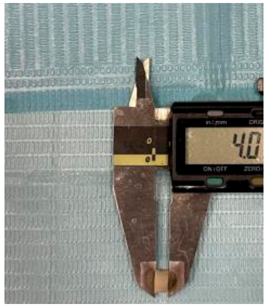	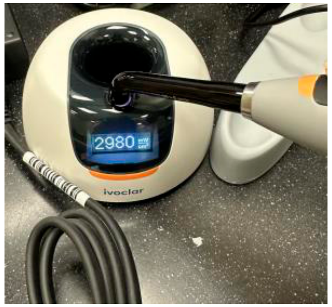
**E. Radiometer output — conventional setting**	**F. Experimental groups overview**	**G. Representative labeled group (SDCL)**	
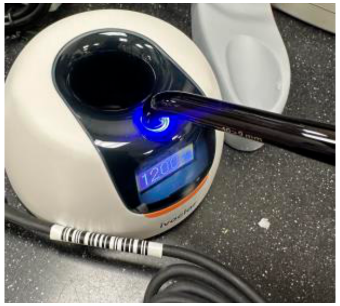	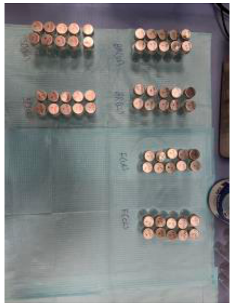	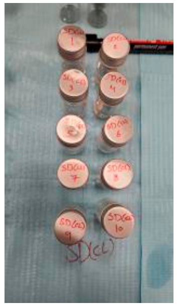	

Figure S1. Representative photographs documenting specimen fabrication, specimen thickness verification, light-output verification for the curing protocols, and the labeled experimental groups. Disk-shaped specimens were prepared using custom molds and allocated according to material and curing protocol.

Figure S2. Representative ATR-FTIR spectra

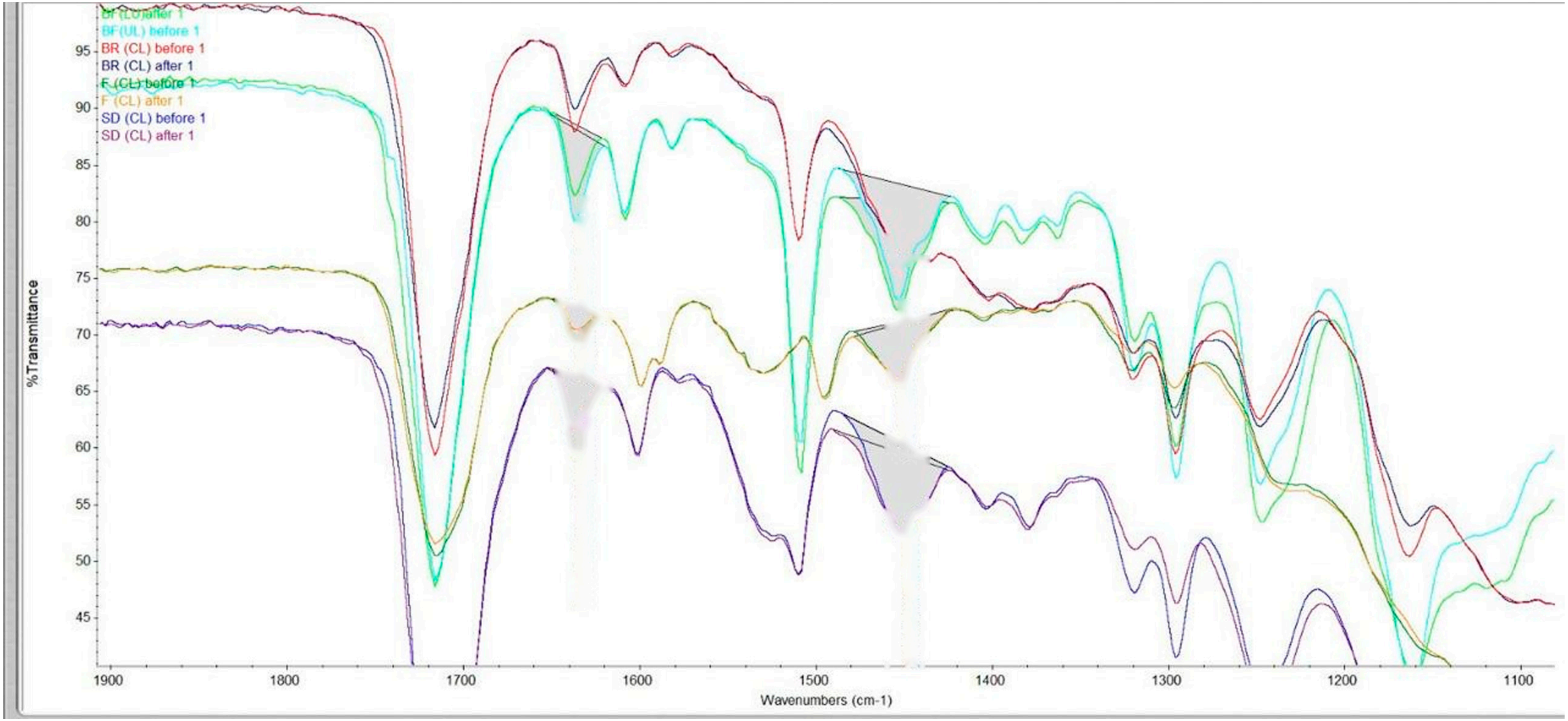

Figure S2. Representative ATR-FTIR spectra of the tested bulk-fill resin composites before and after light curing. The spectra illustrate the characteristic absorption bands used for degree-of-conversion calculation, including the aliphatic C=C band around 1640 cm⁻¹ and the aromatic reference band around 1608 cm⁻¹.

Figure S3A. Knoop indentation images — F and SD groups

	Before ethanol	After ethanol
**FCL**	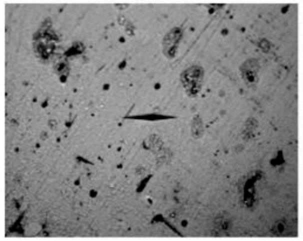	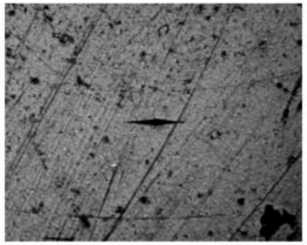
**FUL**	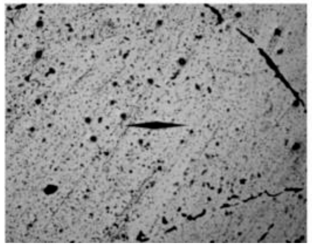	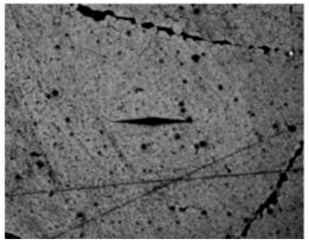
**SDCL**	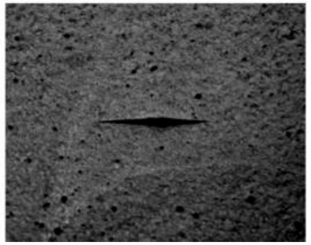	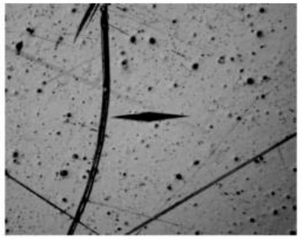
**SDUL**	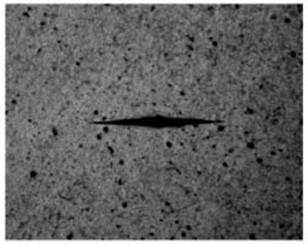	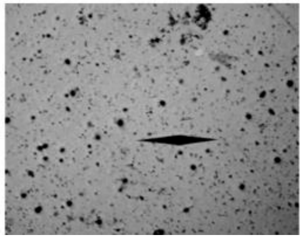

Figure S3A. Representative optical images of Knoop indentations before and after 24 h ethanol immersion. These images support the Knoop microhardness measurements used for the indirect assessment of cross-link density. Images shown are representative; the complete set of indentation records is available.

Figure S3B. Knoop indentation images — BF and BR groups

	Before ethanol	After ethanol
**BFCL**	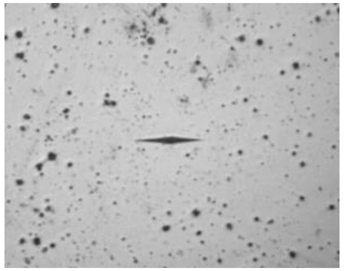	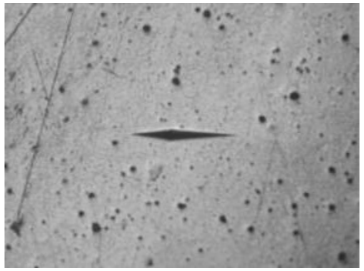
**BFUL**	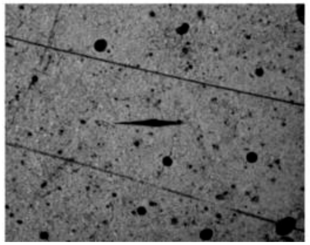	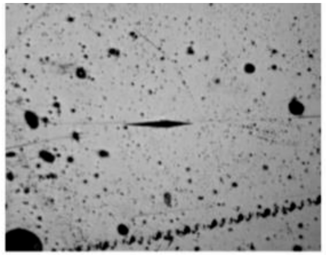
**BRCL**	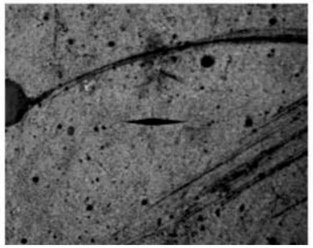	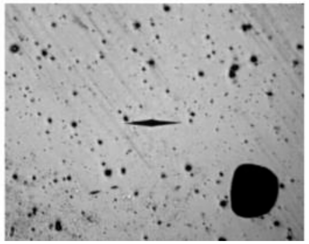
**BRUL**	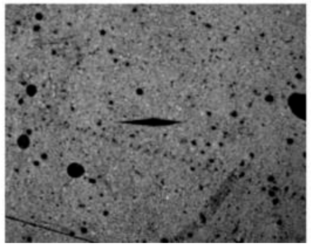	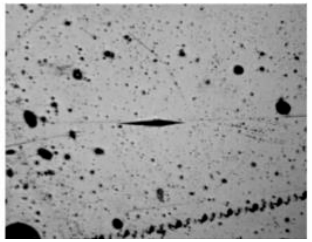

Figure S3B. Representative optical images of Knoop indentations before and after 24 h ethanol immersion. These images support the Knoop microhardness measurements used for the indirect assessment of cross-link density. Images shown are representative; the complete set of indentation records is available.
